# Does it exist pulmonary hypertension in the ELBW infants?

**DOI:** 10.1186/1824-7288-41-S1-A6

**Published:** 2015-09-24

**Authors:** Carlo Dani

**Affiliations:** 1 Department of Neuroscience, Psychology, Drug Research and Child Health, Careggi University Hospital of Florence, Italy

## 

Pulmonary hypertension (PH) of the newborn is a severe complication that occurs more frequently in term infants but can be demonstrated also in preterm infants. The incidence of PH in preterm infants is currently unknown because it is often masked by the contemporary respiratory distress syndrome (RDS), there are not important echocardiography studies in the early transitional period assessingthe presence of PH in this population, and its echocardiographic diagnosis is not always simple. Similarly to term infants, PHin preterm infants can result from an abnormal transition from fetal to neonatal life (persistence of fetal circulation) but more frequently complicates RDS, or is secondary to the abnormal lung growthcaused by maternal pregnancy diseases, such as preterm premature rupture ofmembranes (PPROM) and oligohydramnios or, finally, is associated to bronchopulmonary dysplasia (BPD). On the other hand, it has been recently demonstratedthat early PH occurring in association with severe RDS is a risk factor for late PH and BPD in preterm infants.

The first choice drug for the PH of the newborn is inhaled nitric oxide (iNO), but its effectiveness in preterm infants is highly debated. American Academy of Pediatrics in 2014 reported that previous studies indicate thatneither rescue nor routine use of iNO improves survival in preterm infants with respiratory failure. However, preterm infants with echocardiographic diagnosis of PH have not been specifically evaluated in previous studies and it is possible that theyrepresent a subgroup witha different response. Interestingly, milrinone has been recently found to be effective in improving oxygenation of preterm infants with PH refractory to iNO.

In conclusion, it seems that PH actually exists in extremely low birth weight infants, and that many efforts are needed to improve their finding and their treatment.
